# Metrorrhagia and the Menopause1Presidential address delivered at the twenty-fifth annual meeting of the Alumni Association of the Medical Department of the University of Buffalo, April 27, 1900.

**Published:** 1900-07

**Authors:** William Warren Potter

**Affiliations:** Buffalo, N. Y.


### METRORRHAGIA AND THE MENOPAUSE.[1]

By WILLIAM WARREN POTTER, M. D., Buffalo, N. Y.

TWENTY-FIVE years ago this association held its first public meeting, perfected its organisation, and began its work. These exercises took place in the lower amphitheatre of the old college building, at the corner of Main and Virginia Streets. In the evening it was my privilege to deliver the first public address to the Alumni from the commencement stage in St. James Hall, which stood on a portion of the site of the Iroquois Hotel. It was then the custom to have two addresses at commencement—one to the graduates, and another to the alumni.

The next year I was chosen president of the association, and for several years was a member of the executive committee. Last year it was the pleasure of the association again to call me to the chair, and for all these indications of respect and confidence I beg you to accept my grateful thanks.

A few months ago while in attendance upon a meeting of one of our prominent national special societies I heard a paper read[2] which, with the discussion it evolved, made a deep impression upon me. Not because of its novelty, for it was not new; nor was it because of original thoughts developed in the discussion,—for they were not altogether original,—was I particularly impressed; but rather because of the way the subject was presented, and, further, because of the forceful manner of the discussion with reference to two or three points, did it so attract my attention as to determine the topic of the discourse to which I shall invite your attention for a few moments on this occasion.

---

1. Presidential address delivered at the twenty-fifth annual meeting of the Alumni Association of the Medical Department of the University of Buffalo, April 27, 1900.

2. Hemorrhage and the Menopause, John Milton Duff, Trans. Am. Ass'n Obs. and Gyn., 1899.

The most that I shall expect to do now will be to accentuate those features of the menopause that experience and observation have led me to regard as too important to be dealt with in a trivial manner. The general practitioner or family physician, as a rule, is consulted first by women who have some unusual symptoms developed during the approach of the climacteric. Too often are such patients told that they must expect almost any and every symptom that can be imagined during that mysterious period popularly termed the "change of life." So long as the symptoms can be classed as, strictly speaking, nervous phenomena, no harm may be done by tentative management; but if there be hemorrhages, excessive or recurrent, they may be regarded as of serious portent, and may require the closest diagnostic acumen to determine their cause. At all events, they are *prima facie* evidence of pathological changes that must be inquired into with all the skill that belongs to our art. It is at this time that the beginning of malignancy may be detected if investigation is thorough, and if found there must be no delay in adopting some radical measures of relief. Time was when such a patient was doomed at the outset, and abandoned to a fate more dreadful than death itself. Now, however, thanks to a progressive and enlightened surgery, if a proper and early operation is done a cure may be hoped for in a goodly number of cases, and relief from suffering or distressful conditions in the majority, with prolongation of life in nearly all.

It must be remembered, however, that hemorrhages are not always concomitants of malignant disease, neither is pain, which have always been associated in the minds of non-professional persons, especially when persistent, as indications of cancer. These two symptoms, hemorrhage and pain, have, indeed, even in the estimation of physicians, been regarded as classic symptoms of malignant disease, but cancer has been known to progress even to advanced stages without decided hemorrhage or pain—an exception to the general rule, that must not be overlooked and a favorable diagnosis reached, based upon the absence of these two important signs.

There are other diseases of the genital tract, non-malignant, that may exist with hemorrhage as a prominent symptom. We may recall, for example, that a history of hemorrhage has been associated with the presence of fibroma, uterine cysts, disease of the appendages, and of the endometrium, all of which may occur in women between forty and fifty years of age, be strongly suspicious of malignancy, and yet be absolutely benign in character. So, I observe, that it will not answer to make hasty interpretation of symptoms singly or in groups, but only after duly weighing all phenomena, historic, physical—subjective and objective—can a fixed and definite conclusion be reached as to the proper interpretation to be placed upon hemorrhages at or near the menopause.

In view of all this—and much more might be said in elaboration of this part of the subject—it is safe to assume that every persistent and recurrent hemorrhage occurring in a woman during the fifth decade of life, shall be regarded with suspicion. It is indicative of some important pathologic change; it should receive the most considerate attention by a physician who may be consulted, and he should not stop short of a careful physical examination of a patient so complaining. Such an one should be duly and seriously informed of the significance of such a symptom, and she should submit herself to the most considerate investigation at skilful hands. On the other hand, when a physician is consulted by such a patient he must not regard his responsibility lightly. He must not say that such and such symptoms are to be expected at this time of life, and that they mean very little or nothing. He must not convey the impression to a woman approaching the climacteric with unusual symptoms, that she is unnecessarily concerned about herself, until he is satisfied that such is the fact, after he has most patiently weighed every possible piece of evidence that can be obtained. This means the most thorough examination of the gential tract that modern gynecology can offer, an examination supported if need be by the microscope, or any other accessory that may help to arrive at the truth. It means, too, if malignant disease of the uterus be detected, an immediate hysterectomy, total, complete, absolute—to be done now. Delays here are as dangerous to life as in fulminating appendicitis, and every person, professional and lay, knows that procrastination in such a condition is not to be brooked on any account.

If operation is promptly made in early cases success is almost assured, provided the uterus and appendages are removed. We could not speak as confidently when curettage of the diseased cervix in carcinoma was an accepted plan of procedure. Then it was not easy to remove all invaded structures, some nodules were almost sure to be left behind, and these formed a nidus for a new development of the disease, which almost always then spread rapidly to a fatal issue. Now, with the improved surgery, whereby all infected organs may be removed under perfected technique and asepticism, operation may be more confidently advised,—even insisted upon under favorable conditions. I do not wish to be understood as saying that excision always will cure even in well selected cases, for so much, after all, depends upon systemic states, resisting power, and what may be termed general surgical fortitude in this as well as in most other high-class surgery; but I do affirm that under the favoring conditions of early diagnosis, prompt and complete surgery, localised infection, and good resisting power, many women are cured, who heretofore were lost; many more have their lives considerably prolonged; and still others are relieved of great mental and physical anguish. Taken altogether, then, there has been great improvement in methods of dealing with malignant disease of the uterus during the past twenty years. When this association was organised twenty-five years ago there was little that was encouraging to be said to a woman who presented the evidences of carcinoma of the genital tract. Our methods of recognising the disease in its early stages were less determined, and our methods of operating were far below the present standard.

One of the greatest difficulties we have to contend with in this country is to get patients to pay sufficient heed to their early symptoms. There is a tradition among the people that the climacteric is naturally fraught with perturbation of body as well as spirit, hence many times such patients do not consult physicians for symptoms that need intelligent interpretation. It is said that in some parts of continental Europe, perhaps in Austria and Germany, cancer of the uterus is more successfully dealt with than here, not because there is more professional skill exercised, but in those countries women beset the clinics early, hence receive prompter attention, and remain longer under observation. In America there is less of that spirit prevalent, less opportunity, perhaps, to invoke efficient aid in early manifestations of the disease, and, it may be, more indifference on the part of the great body of the profession toward the hemorrhages of the anteclimacteric period.

In the course of the discussion of this subject, to which I made allusion at the outset of my remarks, it was stated that the general practitioners of medicine who usually meet these patients first were more or less responsible for the delay they experience. It was affirmed that the general practitioner was not always alive to the importance of the situation, that he often regarded hemorrhages as incident to the "change of life," and led his patients to so believe, until finally, when the real condition became apparent it was too late for surgical relief, because the halcyon days were past. Without affirming or denying the statement, I present it for consideration at this time.

We are gathered here today as general practitioners, we mingle as such for the day, no matter what our several specialties in practice may be. The specialist who is not competent to give advice as a general practitioner, is not deserving the confidence of the public as a specialist. To a great extent the converse of this proposition is true.

Again, for the day we are all young men—pardon my pride and modesty in so rating myself—we are for the time being of the class of 1900, and must be permitted to counsel together as such. We are the boys and girls of the U. of B., we revel in our youthfulness, and in the progressive work of our alma mater, who, though 55, is as robust and vigorous as her youngest brood.

Let us then consider whether we, or any of us, are in error in the view we take of this grave subject, and in the advice we give to these who suffer from hemorrhages at or near the menopause.

I blame myself for having made such mistakes in my early years of practice, but none of the men of 1900 can be forgiven if they commit such grievous blunders.

You have been taught as students to regard such symptoms as serious and to so impress your patients. So, too, have your predecessors for fifteen or twenty years. My object in introducing the subject is to accentuate the importance of the teachings you have received regarding it, and to urge you to heed them to the very letter. It is one of the most important duties you can perform to your patients, and you cannot escape censure if it is neglected or performed with indifference.

It is the duty of every alumnus of this college who has graduated within the last twenty-five years to constantly and habitually attend the meetings of his local medical society, and yield the weight of his or her influence in discussing this subject on proper occasions, until there is aroused a sentiment that will serve to change the impression among the laity, that the "change of life" is a time when women must expect hemorrhages. The fact is, that the menopause means the very reverse; it is a time when the physiological flow must cease, and it must not be replaced by a pathological flow if a woman is to be called healthy, or if she is to be told that there is no occasion for anxiety.

These, I am aware, are only generalities. I leave the particulars to be supplied by those who may take part in a free discussion of the subject which I now most cordially invite.

284 Franklin Street.

